# On microbes, aging and the worm: an interview with David Weinkove

**DOI:** 10.1186/s12915-018-0600-x

**Published:** 2018-11-01

**Authors:** David Weinkove

**Affiliations:** Department of Biosciences, Durham University, Stockton Road, Durham, DH1 3LE UK

**Keywords:** Aging, *C. elegans*, Bacteria, Lifespan, Nutrition

## Abstract

David Weinkove is an associate professor at Durham University, UK, studying host–microbe interactions in the model organism *Caenorhabditis elegans*. David has been focusing on the way microbes affect the physiology of their hosts, including the process of aging*.* In this interview, he discusses the questions shaping his research, how they evolved over the years, and his guiding principles for leading a lab.

## What are the questions driving your research?

A key question driving our current research is how do bacteria influence animal physiology, and ageing in particular? Animals originated in a microbial world, so microbes must have shaped animal evolution. Animals defend themselves from microbes, but have also taken advantage of the situation, for example by relying on microbes to synthesise key vitamins. We use the simplified system of the nematode *Caenorhabditis elegans* and the bacteria *Escherichia coli* to try to understand these relationships and how they affect ageing.

More recently I have become interested in two related questions: 1) can ageing be explained and understood as an emergent property of how living systems work? And 2) can we improve our measurement of ageing in *C. elegans* through automation to enable a deeper understanding?

As it is so easy to grow and genetically modify *C. elegans*, I have often wondered, can we use *C. elegans* to make high-value products? We are trying to use *C. elegans* to make a protein that can currently only be isolated from parasitic worms but has the potential to be a therapeutic for autoimmune diseases.
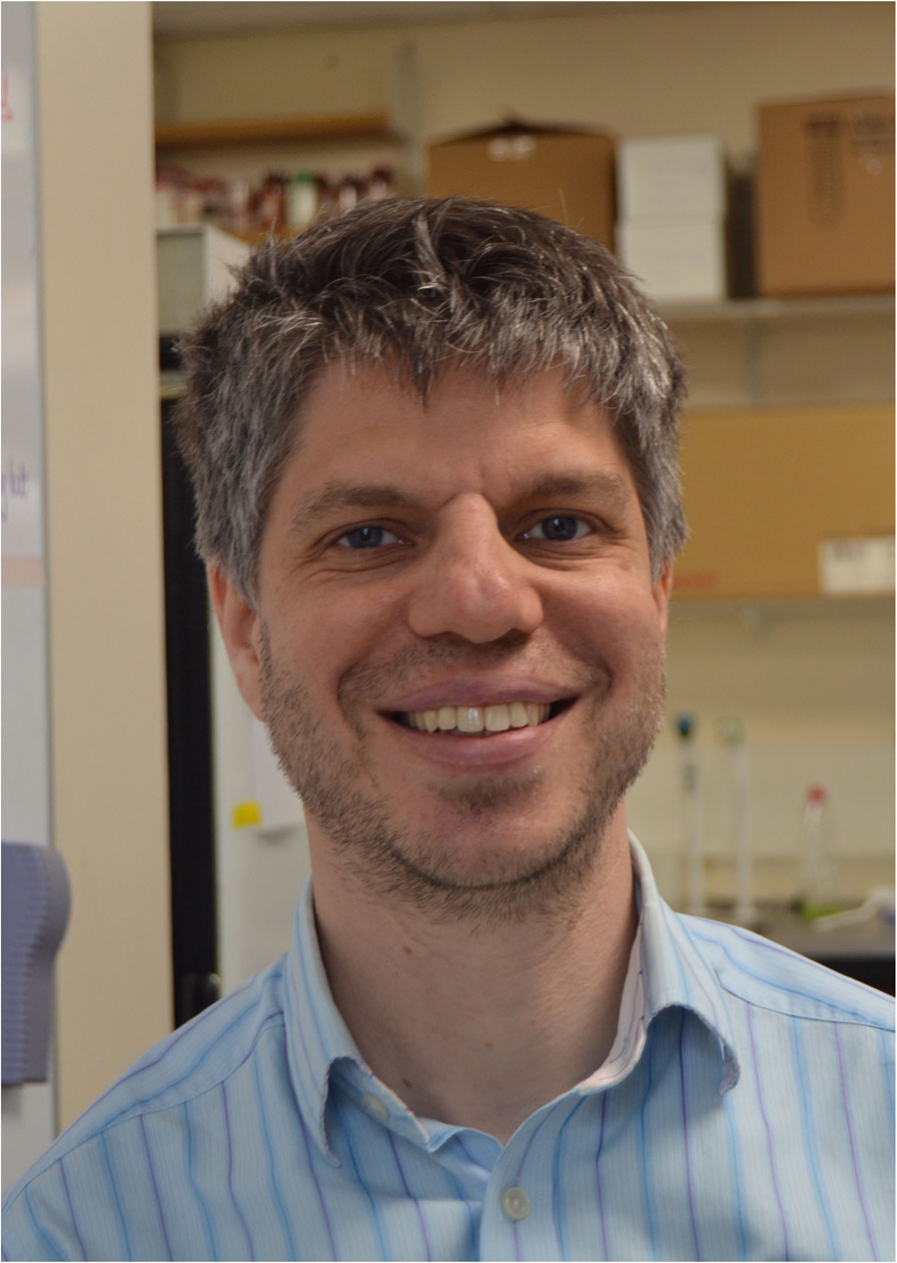


## You published several papers in *BMC Biology*; your first one was while you were a postdoctoral researcher, and the most recent one came out just this year. Can you tell us a little bit how your scientific interests developed over that time?

My first *BMC Biology* paper was about understanding how the *C. elegans* AGE-1 enzyme worked [1]. It was satisfying to combine genetics, biochemistry and microscopy to address this question but the starting point was how does this gene/enzyme work? My scientific questions now usually start with a more “biological question” such as how do bacteria influence ageing? Having said that, my other two *BMC Biology* papers arose from serendipitous observations: a spontaneous mutant of *E. coli* that extended *C. elegans* lifespan and the finding that folic acid needs to go via the bacteria to supplement the worm [2, 3].

## Looking back, is there a project that your lab pursued that stands out for you as particularly inspiring, tough or simply memorable?

It was an amazing experience to follow a strain of *E. coli* that dramatically increased *C. elegans* lifespan and realize that the lifespan increase had nothing to do with the RNA interference plasmid this strain contained but was due to a spontaneous mutation of the *E. coli* chromosome. I identified the mutation through a complementation screen and we found that it increased worm lifespan by impeding bacterial folate synthesis [3]. This discovery led to a large programme of work we are still pursuing [4].

## Is there a paper or a scientist that inspired you, or was seminal for your research?

I worked in ten different labs before starting my own, and I learnt something from all the PIs I worked with and all my other colleagues in those groups.

## What are your guiding principles for running a lab? Do you have any advice to share with our readers?

I think it is important to give members of my group as much autonomy as possible, but this doesn’t mean that they are ignored. I try to give support and autonomy at the same time. In the short term it may be less efficient than directing projects in detail but in the long-term autonomy fosters ownership of the project, motivation and makes for a better, self-sufficient scientist.

## If you could, what would you tell your younger self?

The same thing I tell my older self! “Spell it out”. I find it difficult to remember that the reader isn’t in my head when I’m writing and therefore the layers of thought behind each statement need to be explained. And my younger self would tell my older self “Enjoy your science”, because the fun of science is a great motivator.

## What kind of innovations in publishing would you like to see happen?

Journals paying reviewers. Good peer review is difficult and time-consuming. It should be rewarded, and being paid might make reviewers less grumpy!

**Website:** https://www.dur.ac.uk/biosciences/about/schoolstaff/academicstaff/?id=6328

**Twitter:** @dweinkove
